# Severe injury to the brachial neurovascular bundle and muscles due to a horse bite: a case report

**DOI:** 10.1186/s13256-021-02863-w

**Published:** 2021-05-25

**Authors:** Hajriz Rudari, Luan Jaha, Adhurim Koshi, Lulzim Vokrri

**Affiliations:** Department of Vascular Surgery, University Clinical Center of Kosova, Pristina, Republic of Kosovo

**Keywords:** Horse bite, Arterial injury, Sepsis, Hemodialysis

## Abstract

**Introduction:**

Only a small portion of horse injuries are related to horse bites. In the majority of these occurrences, injuries are minor and self-treated. However, in some cases, the injury may be destructive and limb- and life-threatening. In these instances, the patient requires complex surgery and compound perioperative care.

**Case report:**

We present the case of a 35-year-old Albanian male farm-worker in whom a horse bite caused an extensive lacero-contusive and avulsive wound to the arm. The wound resulted in injury to the brachial artery, brachial and basilic vein, and biceps and brachialis muscles. Nerve structures and underlying humerus remained intact. The initial management of the severe hemorrhagic shock caused by the bleeding at the site of injury included reconstruction of the brachial artery by interposing saphenous graft and that of the brachial vein by termino-terminal anastomosis. Basilic vein was ligated. The wound was extensively debrided, and after a drain was placed in the wound, biceps and brachialis muscles were reconstructed. The patient received several units of red blood cells and fresh frozen plasma before and after surgery, as well as antibiotic, antitetanic, and antirabies prophylaxes. He had several consecutive necrectomies in the following days. However, due to postoperative sepsis and hemorrhagic shock at time of admission, the patient developed acute renal failure, therefore requiring several hemodialysis sessions. After his general and local condition was stabilized, the patient also underwent several reconstructive surgeries.

**Conclusion:**

Horse bites of large extent require a multidisciplinary approach. The composition of the team of physicians needed for treatment varies depending on the degree of the injury and eventual complications. In the case of our patient, emergency department physicians, vascular and plastic surgeons, intensive care specialists, nephrologists, and infective care specialists were involved. In different instances, the inclusion of other specialists may be necessary to save and functionalize the limbs of the patient, or save his/her life.

## Introduction

Animal bites are fairly common. Almost 2% of all emergency admissions are related to animal bites [1], with dog bites being the most frequent (80%–90%), followed by cat and human bites. Horse bites account for only a small portion of animal bites [2]. The head and neck are the most frequent horse bite sites, followed by the extremities and trunk [3]. Injuries to the great vessels, especially those exsanguinating, are very rare.

In the majority of occurrences, horse bite injuries are minor and self-treated. However, in some cases, the injury may be destructive, and limb- and life-threatening. In these instances, the patient will require complex surgery and compound perioperative care. This was the case with our patient in whom a horse bite caused laceration of brachial artery and vein that led to hemorrhagic shock, complicated with infection and renal failure.

## Case report

**Fig. 1.**
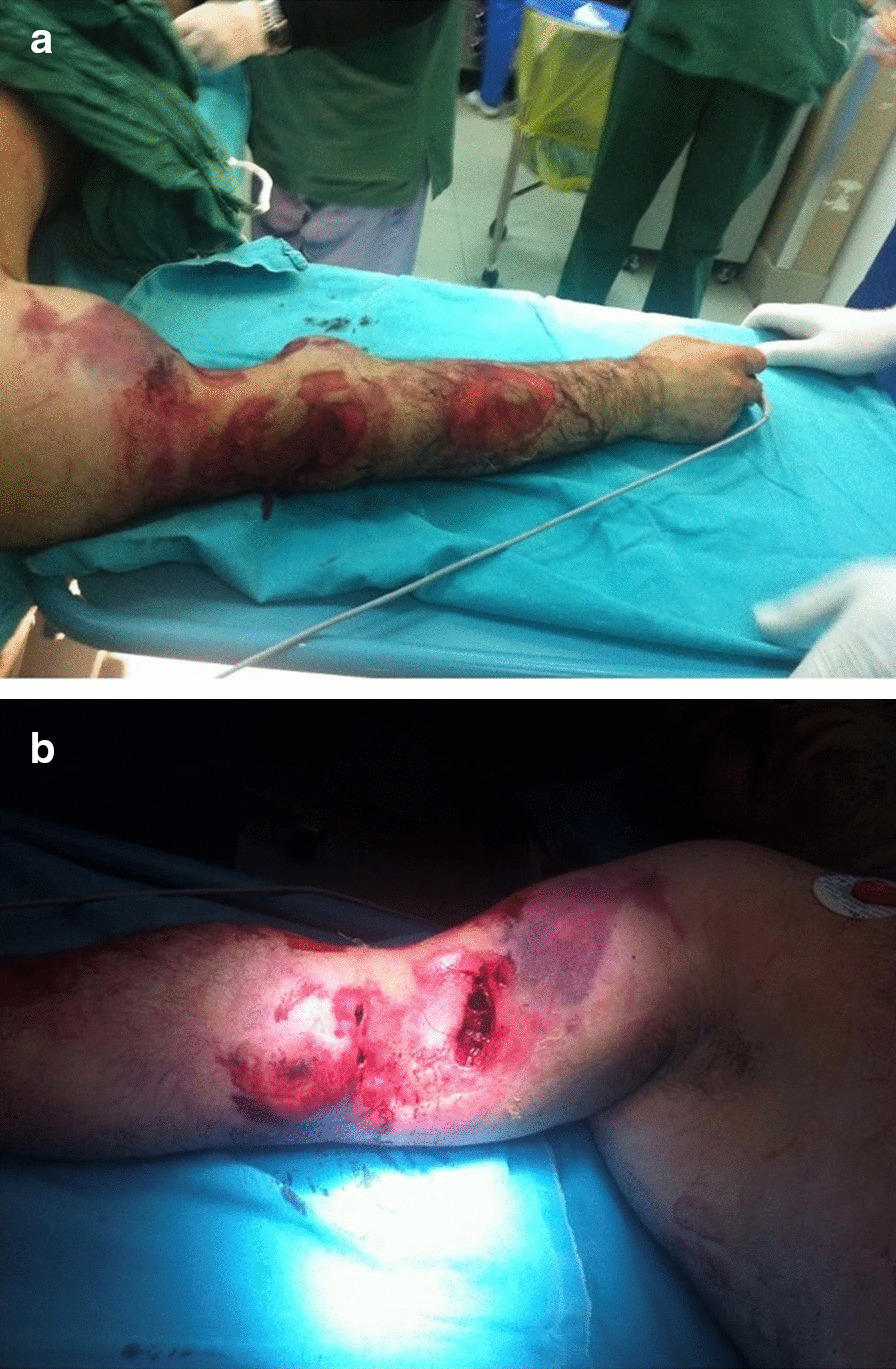
Horse bite injury of right upper extremity: lateral (**a**) and medial (**b**) aspects

A 35-year-old Albanian male farm-worker without relevant medical, physical, or psychosocial history was brought to our emergency room in a state of major hemorrhagic shock with an injury inflicted by a horse bite, just 5 hours prior to admission. His arm was heavily deformed with skin, fascia, and muscles torn from the humerus, which was visible at the bottom of the wound (Fig. 1a, b). Due to thrombosis of the lacerated vessels, there was no bleeding at time of admission. Pulse was missing on the right radial and ulnar arteries, and movement was limited. It was impossible to correctly examine nerve function due to difficulties in communication caused by the state of shock. We covered the wound and started resuscitation.

The patient was confused and anxious, and his skin was pale, cold, and sweaty. He was breathing rapidly. The pulse on his left radial artery was weak, rapid, and thready. Pulse in his right arm was missing. His fingernails and lips were blue, and his capillary refill time was 5 seconds. He had blood pressure of 70/40 mmHg, heart rate of 130 beats/minute, and peripheral capillary oxygen saturation of 92%. His hematocrit was 18%, and his hemoglobin was 6.8 g/dL. His platelet count was normal, and his white blood cell count was slightly elevated (11.0 × 10^9^/L). His glucose, cholesterol, urea, and creatinine concentrations were all within normal range. His total bilirubin was moderately elevated (32.4 μmol/L), and his transaminase level was normal. He had a significantly high level of C-reactive protein (55.8 mg/L). His urine was clear. No serology or microbiology was performed. Due to the urgency of the case, no additional diagnostic evaluation (radiography, duplex scan, angiography, or neurography) was performed. Hemodynamic resuscitation was initiated immediately. Two large-bore intravenous catheters (16-gauge) were inserted. Crystalloids and colloids were administered rapidly, and red blood cells and fresh frozen plasma (FFP) were ordered. Induction agents etomidate (0.3 mg/kg), fentanyl (3 μg/kg), and rocuronium (1.2 mg/kg) were administered. The patient was intubated and escorted to the operating room. Anesthesia was maintained with sevoflurane (0.7–1.3 minimum alveolar concentration), atracurium, and fentanyl. To achieve hemodynamic stability, vasopressors (dopamine 5–7 μg/minute) were used until several units of red blood cells and FFP were brought from the transfusion desk. To minimize the possibility of rebleeding, permissive hypotensive resuscitation was maintained.

**Fig. 2.**
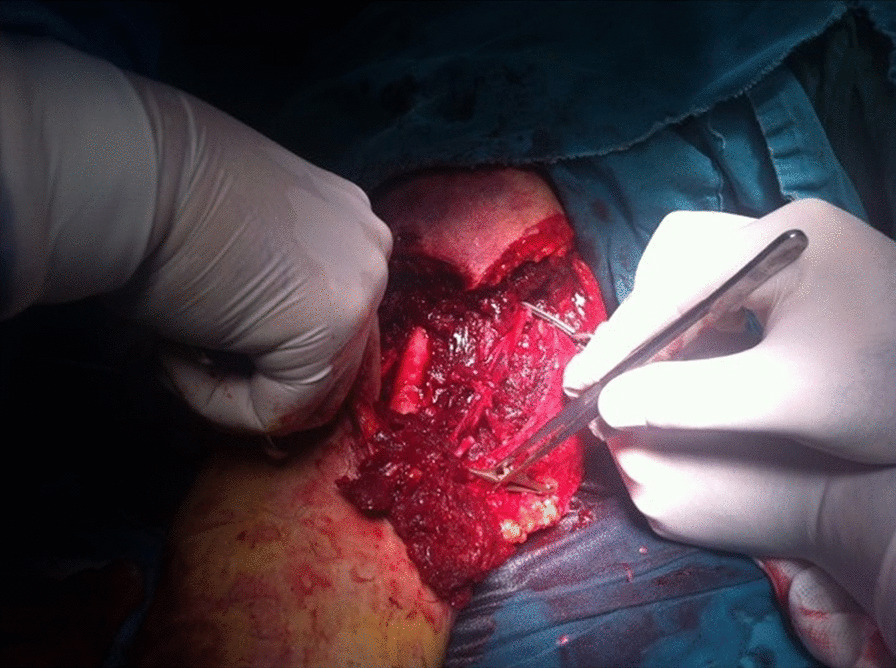
Injured and clamped blood vessels of the arm, brachial artery, and brachial vein

**Fig. 3.**
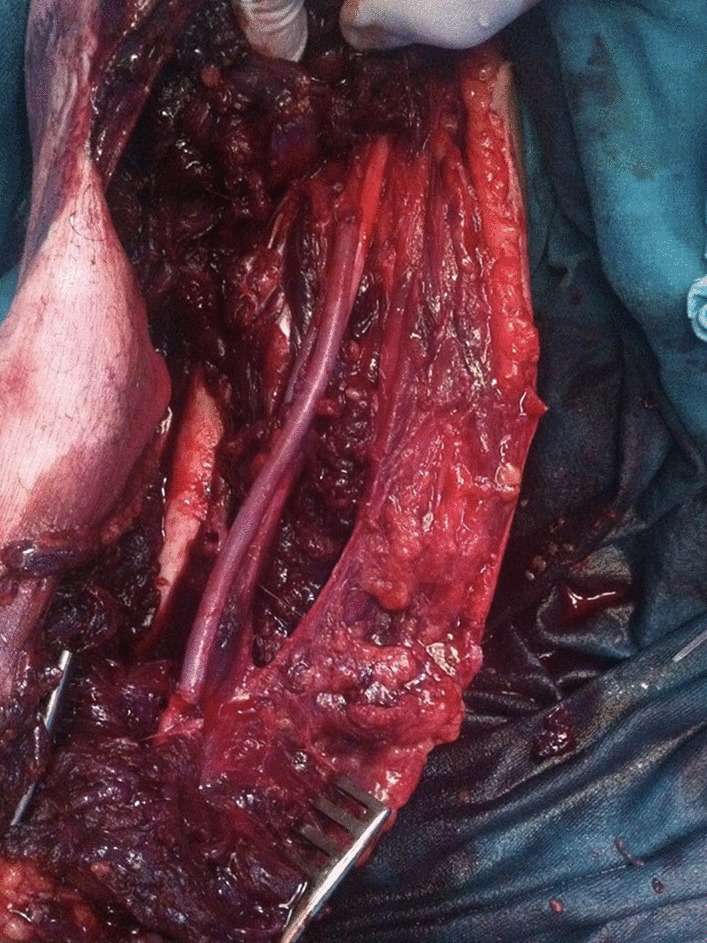
Brachial artery reconstructed with autologous saphenous vein

At the operating table, we found large lacero-contusive and avulsive wounds with parts of biceps and brachialis muscle, fascia, and subcutaneous tissue completely torn from the humerus. After careful debridement, we approached the medial neurovascular bundle of arm, where we found complete laceration of the brachial artery, brachial and basilic vein and intact deep brachial artery, ulnar nerve, brachial nerve, and antebrachial cutaneus nerve (Fig. 2). We dissected these structures and obtained proximal and distal control. Due to extensive laceration of the brachial artery, it was not possible to perform end-to-end anastomosis. This was not the case with the brachial vein, which we were able to mobilize, debride, and reconstruct by end-to-end anastomosis. To reconstruct the brachial artery we used a 10-cm segment of saphenous vein harvested from the leg and implanted it between two ends of the debrided brachial artery (Fig. 3). The lacerated basilic vein was ligated. Afterwards, the muscles were sewn. Finally, a suction drain was placed. One week after surgery, following formation of granulating tissue, autologous skin graft was used to close the wound.

After surgery, patient resuscitation continued. The patient was put on a broad spectrum of intravenous antibiotics (vancomycin and metronidazol), and antitetanus and antirabies prophylaxes. *Enterococcus* was isolated from the wound. The bacteria resulted sensitive to the mentioned antibiotics, thus we continued with the same treatment. The local infection was treated with frequent debridements. However, major blood loss and hemorrhagic shock caused by laceration of the brachial artery, brachial and basilic vein, infection, limb ischemia, and massive destruction of soft tissue of arm and forearm led to complicated postoperative course and caused renal failure. Therefore, the patient underwent several sessions of hemodialysis. Once the treatment was completed, the renal function, as well as the local and general condition of the patient, improved significantly.

**Fig. 4.**
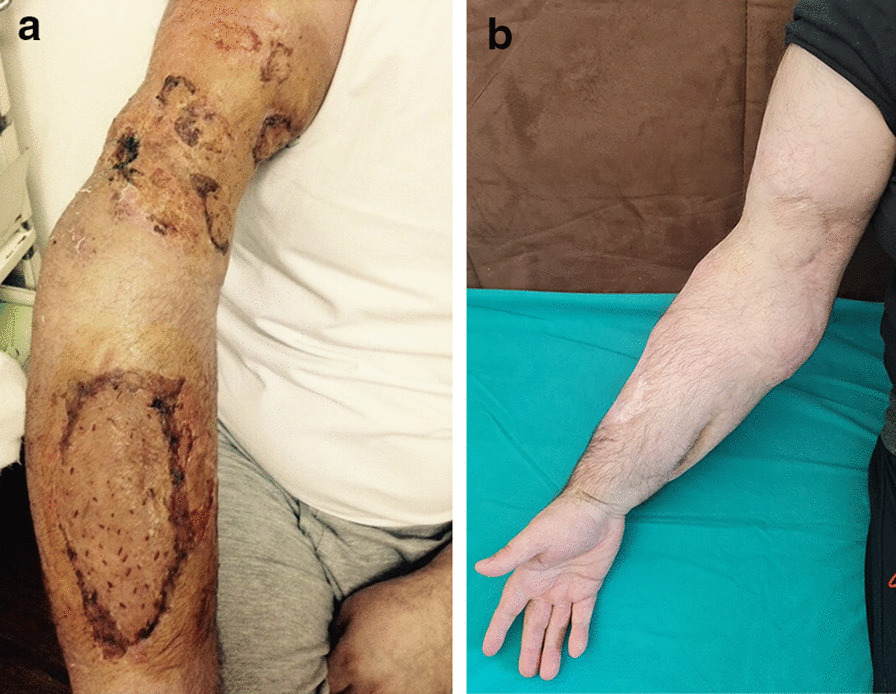
Hand 6 weeks after injury (**a**) and 2 years after operation (**b**)

The patient was discharged 3 weeks after the surgery. Follow-up clinical examination, electoneurography, and electromyogaphy of arm muscles (performed 6 months, 1 year, and 5 years after discharge) showed no signs of sensory or motor deficit (Fig. 4a, b).

## Discussion

The majority of injuries in horse-related accidents occur when a person is blown or kicked by a horse, falls from a horse, gets struck by an object while riding a horse, gets trapped by a horse falling on them, and/or becomes entangled by reins [4, 5]. Only 3–4.5% of these injuries are related to bites [6]. However, although rarely, horses are the animals most commonly involved in bite-related fatalities [2] and are responsible for almost 2% of all emergency admissions [1].

Because of the great deal of force exerted by an equine in closing its jaws, the severity of injuries may range from mild superficial pressure trauma, cutaneous breaks of the skin, deep lacerations with loss of tissue, and injuries to blood vessels and nerves, to amputations of digits or breast, and fractures [7–9]. These wounds are generally contaminated due to the large number of bacteria in the mouth of horse, and their treatment is difficult. Horse bites most commonly lead to infections with *Burkholderia*, *Streptococcus*, *Staphylococcus*, *Rhodococcus*, *Actinobacillus*, *Yersinia*, *Pasteurella*, *Escherichia*, *Neisseria*, *Prevotella*, *Pseudomonas*, *Listeria*, and *Hendra* and *Vesicular stomatitis* virus species [10–12]. The wound in our patient was infected with *Enterococcus*.

Among horse-related injuries, fractures have been reported mainly following falling accidents [1]. Peel et al. [13] reported a case of fracture of the forearm bones following a horse bite that was treated with open reduction and internal fixation primarily. The patient sustained repeated infections with purulent wound discharge, from which mixed cultures of bacteria, including *Staphylococcus aureus*, *Prevotella melaninogenica*, *Escherichia coli*, and *Pasteurella multocida*, were isolated. More than 3 months after the initial attack by the horse, *Actinobacillus suis* was isolated by bone biopsy specimen.

The occurrence of bacterial infection after animal bites depends on several factors, such as the species of the animal aggressor (humans being associated with a higher risk of infection), the type and site of the injuries (wounds located on the hands have a higher infection risk), the care given to the wound, and factors inherent to the individual (greater risk in elderly, those with diabetes mellitus, vascular disease, etc). In relation to the type of wound, puncture wounds have been reported to have a higher infection rate after animal bites, possibly due to the deposition of bacteria deep into the skin [14]. There is evidence that use of prophylactic antibiotics after horse bites reduces infection [5, 15, 16].

The presented case constitutes a clear example of both high severity of injury and postoperative complications. The horse bite caused laceration of the brachial artery, and the brachial and basilic vein, consequently leading to major blood loss and hemorrhagic shock. Limb ischemia, massive destruction of soft tissue of arm and forearm, and infection in postoperative period, accompanied by prior hemorrhagic shock, led to renal failure and hemodialysis. Both occurrences were identified as limb- and life-threatening to the patient.

The treatment of the patient included the use of autologous vein graft for vascular reconstruction and thorough care for the injured wound, with frequent debridement and necrectomies. Together with intravenous antibiotics and several hemodialysis sessions, the treatment led to patient recovery. Furthermore, reconstructive plastic surgery helped the appearance of the arm to return to a near-normal state.

## Conclusions

Horse bites may cause severe injury to soft tissues and bones due to the strong force applied in the closing jaws of horses. The severity of these injuries can also be aggravated by the heavy load of different pathogenic bacteria in the secretion from the mouth of the horse and extensive destruction of soft tissues and ischemia. In certain cases, the combination of these negative factors can lead to severe infection and sepsis, therefore resulting in failure of kidney and other organs.

To prevent these complications, treatment of horse bite injuries requires extensive debridement of the wound, prompt revascularization, and broad-spectrum intravenous antibiotics. Prophylaxis against tetanus and rabies should also be provided. Only after the local and general condition of the patient become stable should plastic reconstructive surgery be provided. In case of extensive tissue loss and sepsis, hemodialysis may be required.

The composition of the team of physicians needed for treatment varies depending on the degree of injury as well as eventual complications. In the presented case, emergency department physicians, vascular and plastic surgeons, intensive care specialists, nephrologists, and infective care specialists were involved. In different instances, inclusion of other specialists may be necessary to save and functionalize the limbs of the patient, or save his/her life.

## Data Availability

The data are available under consideration of the corresponding author on reasonable request.
